# Occurrence of *Blastocystis* sp*.* and *Pentatrichomonas hominis* in sheep and goats in China

**DOI:** 10.1186/s13071-018-2671-5

**Published:** 2018-02-17

**Authors:** Wen Chao Li, Kai Wang, Youfang Gu

**Affiliations:** College of Animal Science, Anhui Science and Technology University, Fengyang, 233100 China, People’s Republic of China

**Keywords:** *Blastocystis* sp*.*, *P. hominis*, Sheep, Goats, China

## Abstract

**Background:**

Global data regarding the molecular epidemiology of *Blastocystis* sp. and *Pentatrichomonas hominis* in sheep and goats are sparse. China has one of the largest sheep and goat populations in the world. In this study we investigated the occurrence of *Blastocystis* sp*.* and *P. hominis* in domestic sheep and goats in China, and analyzed the genetic characterization of these two parasite species.

**Methods:**

In total, we collected fresh fecal samples from 832 sheep and 781 goats located on seven and ten farms, respectively, in the central eastern region of China. The corresponding sequences obtained in this study were subject to molecular analysis for subtype and allele identification of *Blastocystis* sp., and species and genotype confirmation of *P. hominis*.

**Results:**

The occurrence of *Blastocystis* sp. was 6.0% (50/832) in sheep and 0.3% (2/781) in goats. The most predominant subtype (ST) of *Blastocystis* sp*.* in sheep was ST10 (50.0%), followed by ST14 (20%), ST5 (16%), novel sequence 1 (6%), novel sequence 4 (4%), novel sequence 2 (2%) and novel sequence 3 (2%). However, only ST1 was observed in goats. No mixed infections with different subtypes were found in this study. The 18S alleles showed allele 2 (100%) for ST1; allele 115 (75%) for ST5; and no match allele for ST5 (25%), ST10 (100%), ST14 (100%), novel sequence 1 (100%), novel sequence 2 (100%), novel sequence 3 (100%), and novel sequence 4 (100%) on the *Blastocystis* subtype (18S) and Sequence Typing (MLST) database. For *P. hominis*, two goats (0.3%) and zero sheep (0%) were identified as positive in this study. The 18S rRNA gene sequences of two *P. hominis* isolates from goats displayed 100% identity to type CC1, found previously in dogs, monkeys and humans.

**Conclusions:**

These results provide the detailed data on the occurrence and molecular epidemiology of *Blastocystis* sp*.* and *P. hominis* in sheep and goats in China. They also contribute to and expand our knowledge of the *Blastocystis* sp*.* and *P. hominis* epidemiology around the world.

**Electronic supplementary material:**

The online version of this article (10.1186/s13071-018-2671-5) contains supplementary material, which is available to authorized users.

## Background

Domestic animals (specifically sheep and goats) are prone to several protozoan gastrointestinal infections, with *Cryptosporidium* spp., *Giardia duodenalis* and *Enterocytozoon bieneusi* all being of significant concern to both animal and human health [1]. Recently, increasing awareness has developed regarding the widespread prevalence of the *Blastocystis* species and trichomonad species as one common trend emerging in human as well as animal hosts [2–7].

The anaerobic unicellular eukaryote *Blastocystis* sp. is one of the most prevalent intestinal parasites found in a vast array of host species including humans [2, 8, 9]). The pathogenicity of *Blastocystis* remains both unclear and controversial [7, 10, 11]. Some studies suggest that the presence of the organism might be associated with a number of different diseases including inflammatory bowel disease, irritable bowel syndrome, autism and urticaria [2, 7, 12]. However, recent microbiome and metagenomics studies suggest that *Blastocystis* colonization may be beneficial for human health [7, 10]. Remarkable genetic variation has been described among *Blastocystis* sp*.* isolates colonizing humans, other mammals, and birds. Consequently the genus *Blastocystis* is currently divided into at least 17 distinct subtypes (STs) based on the phylogeny of the small subunit rRNA gene [13, 14]. The STs 1–9 and 12 can infect both humans and animals, whereas STs 10, 11, 13–17 have only been detected in animals [7]. Several studies have shown a higher risk of *Blastocystis* sp*.* infection in humans with close animal contact, as well as identical or high similarity STs between humans and in-contact animals. These findings strongly support the zoonotic potential of the parasite [15, 16].

Trichomonads are amitochondrial anaerobic flagellated protists which possess between three and five anterior flagellas, hydrogenosomes, a parabasal body and a complex cytoskeleton [5]. With the main exceptions of *Trichomonas vaginalis*, *Tritrichomonas fetus* and *Trichomonas gallinae* which are the known etiologic agents of human, cattle and avian trichomonosis, respectively, other trichomonad species like *Pentatrichomonas hominis* are generally believed to be commensal [17–19]. However, studies have shown that *P. hominis* may be the causative agent of diarrhea in humans, pigs, dogs, monkeys, rats and cats [17–19], and may be associated with respiratory tract infections and rheumatoid arthritis in humans [19–23]. The presence of *P. hominis* in new hosts such as boa, scops owl, goats, water buffalo and pigs indicates that this trichomonad may have adapted to new hosts, from mammals to reptiles and birds [6, 24, 25]. Several new studies have highlighted the potential of zoonotic transmission of *P. hominis* between humans and other animal hosts [5, 26].

Despite the potential public health impact and significance of the parasites *Blastocystis* sp*.* and *P. hominis* as potential pathogens in humans and animals, no epidemiological study of the two parasites (except the study on goat *Blastocystis* in Shaanxi province by Song et al. [27]) in sheep and goats is available so far in China. The role of sheep and goats in potential zoonotic transmission of the two parasites is unclear. Therefore, the present research was undertaken to determine the occurrence of *Blastocystis* sp*.* and *P. hominis* in domestic sheep and goat populations in China, and explore the genetic characterization of the corresponding parasite isolates.

## Methods

### Specimen collection

**Fig. 1:**
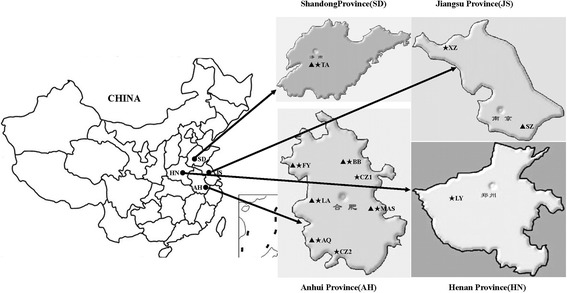
Geographical distribution of sample locations in this study. Triangles indicate the geographical locations at which sheep specimens were collected in this study; stars indicate the geographical locations at which goat specimens were collected in this study. *Abbreviations*: AQ, Anqing; BB, Bengbu; CZ1, Chuzhou; CZ2, Chizhou; FY, Fuyang; LA, Liuan; LY, Luoyang; MAS, Maanshan; SZ, Suzhou; TA, Tianan; XZ, Xuzhou

From September to December 2015, fresh fecal samples were obtained from 832 sheep and 781 goats, from 7 sheep farms and 10 goat farms in the central eastern region of China. Sheep samples were collected from Liuan, Fuyang, Bengbu, Anqing and Maanshan counties in Anhui Province, Suzhou county in Jiangsu Province, and Taian county in Shandong Province. Goat samples were collected from Liuan, Fuyang, Chuzhou, Bengbu, Anqing, Chizhou and Maanshan counties in Anhui Province, Xuzhou county in Jiangsu Province, Luoyang county in Henan Province, and Taian county in Shandong Province (Fig. 1). Sheep and goats in these farms were housed in groups of 10–20 animals within a domed greenhouse-like building. Permission was obtained from farm owners before collection of fecal samples. Both sheep and goats were divided into three age groups: < 6 months (sheep, *n* = 156; goats, *n* = 128), 6–12 months (sheep, *n* = 236; goats, *n* = 247) and > 12 months (sheep, *n* = 440; goats, *n* = 406). Fresh fecal samples were collected either directly from the rectum or from the ground if the animal was observed to defecate. Sterile disposal PE gloves were used to collect the samples. Particular care was taken to avoid environmental contamination. All specimens were marked with the individual’s age and geographical origins, submitted immediately to the laboratory. The stools were stored at 4 °C until DNA extraction, generally within 24 h.

### DNA extraction

DNA was extracted from approximately 200 mg of each fecal specimen using the Stool DNA Kit (Tiangen, Beijing, China), according to the manufacturer’s instructions. The DNA was eluted in 50 μl of elution buffer (Tiangen) and stored at -20 °C until analysis.

### PCR analysis of *Blastocystis* sp*.* and *P. hominis*

In the case of *Blastocystis*, each DNA sample was detected by PCR amplification of *Blastocystis*-specific 18S rDNA using the primers RD5 and BhRDr [28] as recently recommended [29]. For the detection of *P. hominis*, a single-tube nested PCR amplifying a 339 bp sequence of the 18S rRNA gene was performed on each DNA sample [27].

### Sequence analysis

The target PCR products were purified and directly sequenced on both strands. For *Blastocystis*, sequences were edited in BioEdit 7.1 (http://www.mbio.ncsu.edu/ BioEdit/bioedit.html) and compared with reference representing each ST in GenBank using BLASTn (http://www.ncbi.nlm.nih.gov/BLAST). Identification of alleles and ST confirmation were performed by sequence query in the *Blastocystis* 18S database (http://pubmlst.org/blastocystis/). For *P. hominis*, sequences analyzed and aligned with *Tritrichomonas* reference sequences using BioEdit 7.1 and neighbor-joining trees were constructed using genetic distances from the Kimura-2 parameter model in Mega 6.06 (http://www.megasoftware.net/). The reliability of cluster formation was evaluated using a bootstrap analysis of 1000 iterations. Representative sequences generated in this study were deposited in the GenBank database under accession numbers MF991103–MF991111 (*Blastocystis* sp*.*) and MF991102 (*P. hominis*).

### Data analysis

Statistical analyses were performed with SPSS for Windows (release 13.0 standard version, SPSS Inc., Chicago, Illinois, USA). A chi-square test was used to compare infection rates between age groups. Differences were considered statistically significant when *P* < 0.05.

## Results

### Occurrence of *Blastocystis* sp. and *P. hominis*

A total of 50 samples (6.0%) of the 832 sheep specimens were found to be positive for *Blastocystis* sp. by barcoding PCR. The highest percentage was observed in Suzhou County of Jiangsu Province (24%) followed by Taian County of Shandong Province (16.7%) and Bengbu County of Anhui Province (6.4%). There were no significant associations between infection and the collection site locations (Additional file 1: Table S1). Similarly, *Blastocystis* sp. was detected by barcoding PCR in 2 (0.3%) of the 781 goat specimens. *Blastocystis* sp. positive samples were only located in Maansha County of Anhui Province (Additional file 1: Table S1).

*Pentatrichomonas hominis* was only identified in two (0.3%) of the 781 goat specimens, and both positive samples were from the Maansha County of Anhui Province. In sheep no *P. hominis* infection was observed (Additional file 1: Table S1). Infections with both *Blastocystis* sp. and *P. hominis* in the same sample were not observed in this study.

### Distribution of *Blastocystis* sp. STs/alleles and *P. hominis* genotypes

**Fig. 2:**
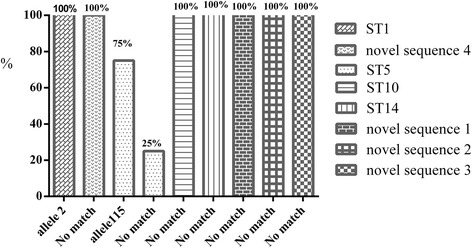
Distribution of *Blastocystis* 18S alleles in sheep and goats surveyed in this study

Barcoding DNA sequencing indicated the presence of three known subtypes (ST 5, 10, 14) and four sequences did not match any in the *Blastocystis* 18S database (temporarily named as novel sequences 1/2/3/4) (Additional file 1: Table S1). The most commonly identified subtype of *Blastocystis* sp*.* in sheep was ST10 (50.0%), occurring in all three *Blastocystis* sp.-positive sheep farms, followed by ST14 (20%), ST5 (16%), novel sequence 1 (6%), novel sequence 4 (4%), novel sequence 2 (2%) and novel sequence 3 (2%). In goats, only one single ST was found in Maansha County of Anhui Province, ST1 (100%). No mixed infections with different subtypes were found in this study. Regarding alleles retrieved from the *Blastocystis* 18S database, for ST1 (allele 2) one allele was detected; for ST5, 75% (6/8) was allele 115, and 25% (2/8) had no match with allele on the database. In the case of samples typed as ST10, ST14, and novel sequences 1/2/3/4, no match (100%) was found on the database (Fig. 2).

**Fig. 3:**
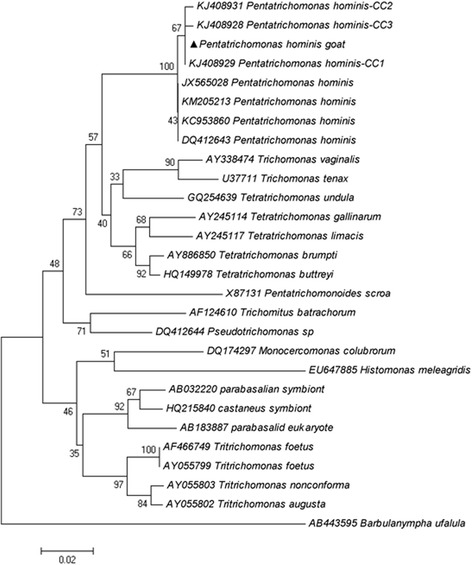
Phylogenetic relationships based on *P. hominis* 18S rRNA gene. Triangle indicates the *P. hominis* isolate of the present study

Sequencing analyses of the 18S rRNA gene in two *P. hominis* isolates identified in this study displayed 100% identity to type CC1 (GenBank: KJ408929). Neighbor-joining trees clearly showed the sequence obtained from fecal specimens belonged to *P. hominis* (Fig. 3).

### Age distributions of *Blastocystis* sp. and *P. hominis*

The occurrence of *Blastocystis* sp*.* and the prevalence *P. hominis* across different age groups is presented in Table 1. *Blastocystis* sp*.* was observed in all three age groups of sheep but only in goats over 12 months old. Among these *Blastocystis* sp*.* from sheep, ST10 was found in all three age groups and ST5 was identified in < 6 month-old and > 12 month-old groups. *P. hominis* was detected in < 6 month-old and > 12 month-old goats. Age was not found to significantly affect the occurrence of *Blastocystis* sp. and *P. hominis* infections.

**Table 1:** Occurrence of *Blastocystis* sp. and *P. hominis* in sheeps and goats in China by age

Host	Age (months)	No. of specimens	*Blastocystis* sp.		*P. hominis*	
			No. of positives (%)	Subtypes (*n*)	No. of positives (%)	Genotype
Sheep	< 6	156	10 (6.4)	ST5(3), ST10(4), novel sequence 1 (3)	0 (0)	–
	6–12	236	13 (5.5)	novel sequence 4 (2), ST10 (1), ST14 (10)	0 (0)	–
	>12	440	27 (6.1)	ST5 (5), ST10(20), novel sequence 2 (1), novel sequence 3 (1)	0 (0)	–
Goat	< 6	128	0 (0)	–	1 (0.8)	CC1
	6–12	247	0 (0)	–	0 (0)	–
	>12	406	2 (0.5)	ST1(2)	1 (0.2)	CC1

## Discussion

The Chinese sheep and goat industry have been the largest in the world since the 1990s, and its proportion in the farming sector is also increasing in China. Sheep and goats are raised traditionally within the family unit in China, and so they have close ties to humans. However, they may be reservoirs of human pathogens. Considering the low host specificity of the two protists, *Blastocystis* sp. and *P. hominis*, transmission between humans and livestock such as sheep and goats warrant serious attention. To our knowledge, the present study represents the first successful attempt to determine the occurrence as well as the genetic diversity of *Blastocystis* sp. and *P. hominis* of sheep and goats in China. The published information globally regarding the molecular epidemiology of *Blastocystis* sp. and *P. hominis* in sheep and goats is limited. This study provides a comprehensive account on occurrence of these two intestinal protozoans among sheep and goats.

The occurrence of *Blastocystis* sp. observed in the present study was 6.0% in sheep, lower than that reported in the UK (23.5%; 12/51) but higher than in Italy (0%; 0/2) [13]. Only two goats (0.3%) were found to be infected by *Blastocystis* sp*.* in our sample population, which was considerably lower than that reported in Malaysia (30.9%, 73/236) [30] and China (58.0%, 458/789) [27]. Furthermore, the occurrence of *Blastocystis* sp. in sheep and goats identified in this study was much lower compared with other livestock such as pigs (7.5–100%), cattle (9.6–80%), and ducks (56%) [31–34]. Statistical analysis implied that host age had no significant effect on *Blastocystis* infection. Our data are inconsistent with that reported by Navarro et al. [35] but are in agreement with the findings of Tan et al. [30]. Infection rates are related to many factors: examination methods, age, sample size, seasonality, to name just a few. Therefore, we are unable to provide a satisfactory explanation for the actual discrepancies in the occurrence of *Blastocystis* sp*.* between different studies.

In this study, *Blastocystis* DNA barcoding was performed revealing the presence of ST1 in goats and STs 5, 10, 14, and four novel sequences in sheep. The novel sequences 1/2/3/4 in this study did not match any STs or allele when submitted to sequence queries at the *Blastocystis* 18S database. Additionally, the four novel sequences were all identified as *Blastocystis* 18S rDNA but displayed at least 4% genetic difference from other STs available in the GenBank by BLAST queries. From experience, the level of genetic difference between STs is at least 4–5%. However, the incompleteness of the *Blastocystis* 18S database (the last data update of the database was done on 16 February 2012) and the barcode region of some *Blastocystis* STs such as ST11 and ST12 were not available on GenBank, thus preventing direct subtyping of the four non-matching sequences. Hence, we did not know whether they are new subtypes or not and named these temporarily as novel sequences 1/2/3/4. The results showed a large diversity of STs, which was in accordance with observations from studies of human and non-human primate populations [14, 36]. ST5, which is the dominant subtype affecting hoofed animals such as pigs and cattle worldwide, was only identified in sheep in Jiangsu Province in the present study [14, 33, 34, 37, 38]. ST5 has also been identified episodically in humans with close animal contact, suggesting zoonotic transmissions [39, 40]. The other zoonotic subtype detected in our study, ST1, has been reported as the most common subtype in humans in many countries [38]. Conversely, although ST10 has never been found in humans, it is very common in livestock [13, 38]. Our results also showed that ST10 was the most predominant *Blastocystis* sp. ST in sheep in this study. In addition, the fact that ST10 was detected in all three *Blastocystis* sp*.*-positive sheep farms in this study supports a previous conclusion that the ST10 distribution is not restricted to certain geographical locations [13]. So far, ST14 was only identified in artiodactyl animals including camels, cattle and mouflons [13, 41, 42]. The present study found that ST14 can also infect sheep. Lastly, novel sequences 1/2/3/4 with different sequence characters, were detected in Bengbu County in Anhui Province and Taian County in Shandong Province. Further studies would determine whether these represent new STs. When the results of the 18S alleles for each ST were retrieved, we observed that ST5 possessed one known allele and no matched allele and ST1 identified in this study had only one known allele. ST10, ST14 and novel sequences 1/2/3/4 showed no matched alleles with those available on the current databases. Allele 2 from ST1 has already been described in Brazil and Colombia; it has a frequency of 55% across South America [10, 32, 43]. Allele 115, is a novel finding: there have been no previous reports of the allele globally.

Interestingly, this study did not identify any mixed infections with several different subtypes. This disagrees with the high frequency (41.1%) of mixed infections of *Blastocystis* detected in goats in Malaysia [30]. A review of the data collected from various surveys across the globe strongly demonstrates that mixed ST infections are uncommon in humans and animals: probably < 10% of all cases [9, 35, 44]. A higher prevalence of mixed infections may be possible to identify with other methods such as the sequence-tagged-site (STS) method [45]. Compared with STS, the barcoding method used in the current study has many advantages, which led us to select it [29, 38]. It should be noted that genus-specific primers (RD5/BhRDr) may cause amplification of other non-*Blastocystis* eukaryotes [9, 38].

Only two recent reports have documented that goats may be a new host for *P. hominis* [24, 25]. To date, it remains unclear whether sheep can become infected with *P. hominis*. In our study, two goats (0.26%) were found to be infected by *P. hominis* and this supported the conclusion that *P. hominis* can infect goats [24, 25]. *Pentatrichomonas hominis* commonly inhabits the large intestine of diarrheic hosts and the liquid or semiliquid anaerobic environment created by diarrhea may provide favorable conditions for opportunistic overgrowth [46]. Goat feces are usually dry and granulated. The stools are seldom liquid or semiliquid even if diarrhea occurs. So this makes it difficult for *P. hominis* to grow and reproduce. This may be one of the reasons for the low occurrence of *P. hominis* infection in goats in our study. The 18S rRNA gene sequence alignment of two *P. hominis* isolates from goats in this study showed a 100% identity and both belonged to type CC1, as described previously by Li et al. [27]. This implies that the two *P. hominis* isolates in this study belong to the same species as those *P. hominis* strains from different hosts [27, 46]. However, all samples from sheep were negative for *P. hominis* in the present study. This may indicate that sheep are not the natural hosts for *P. hominis* and that further studies are needed to clarify the question.

## Conclusions

This present detailed study provided new insights into the molecular epidemiological data regarding the parasites *Blastocystis* sp. and *P. hominis* in sheep and goats in China. Although the observed prevalence of *Blastocystis* sp. was extremely low, these findings indicate that sheep and goats may be a source of zoonotic subtypes of *Blastocystis* sp. The obtained data about *P. hominis* occurrence in sheep and goats provide useful information regarding natural hosts of *P. hominis*.

## Additional file

Additional file 1: Table S1. Occurrence and subtypes distributions of *B. hominis* and *P. hominis* in sheeps and goats in China. (DOC 94 kb)
