# Supplementary material for: Occurrence of Blastocystis sp. and Pentatrichomonas hominis in sheep and goats in China
Source: Parasit Vectors. 2018 Feb 17;11:93. doi: 10.1186/s13071-018-2671-5 (PMC5816562; doi:10.1186/s13071-018-2671-5)
Supplement: Supplementary file 1 — Occurrence and subtypes distributions of B. hominis and P. hominis in sheeps and goats in China. (DOC 94 kb) [file 13071_2018_2671_MOESM1_ESM.doc]

Additional file 1: Table S1 Occurrence and subtypes distributions of *B. hominis* and *P. hominis* in sheeps and goats in China

| **Host and geographic locations** | | | **No. samples** | ***B. hominis*** | | | | | | | | | |  | ***P. hominis*** | | |
| --- | --- | --- | --- | --- | --- | --- | --- | --- | --- | --- | --- | --- | --- | --- | --- | --- | --- |
|  |  |  |  | **No. positives** | **Positive rate/%** | **%****ST1** | **%****ST5** | **%ST10** | **%ST14** | **% Novel sequence 1** | **% Novel sequence 2** | **% Novel sequence 3** | **% Novel sequence 4** |  | **No. positives** | **Positive rate/%** | **Genotypes** |
| Sheep | Anhui | Liuan | 33 | 0 | 0 | 0 | 0 | 0 | 0 | 0 | 0 | 0 | 0 |  | 0 | 0 | – |
|  |  | Fuyang | 111 | 0 | 0 | 0 | 0 | 0 | 0 | 0 | 0 | 0 | 0 |  | 0 | 0 | – |
|  |  | Bengbu | 345 | 22 | 6.4 | 0 | 0 | 63.6 | 13.6 | 13.6 | 4.5 | 4.5 | 0 |  | 0 | 0 | – |
|  |  | Anqing | 108 | 0 | 0 | 0 | 0 | 0 | 0 | 0 | 0 | 0 | 0 |  | 0 | 0 | – |
|  |  | Maanshan | 100 | 0 | 0 | 0 | 0 | 0 | 0 | 0 | 0 | 0 | 0 |  | 0 | 0 | – |
|  | Jiangsu | Suzhou | 75 | 18 | 24.0 | 0 | 44.4 | 27.8 | 27.8 | 0 | 0 | 0 | 0 |  | 0 | 0 | – |
|  | Shandong | Taian | 60 | 10 | 16.7 | 0 | 0 | 60 | 20 | 0 | 0 | 0 | 20 |  | 0 | 0 | – |
| Total | | | 832 | 50 | 6.0 | 0 | 16 | 50 | 20 | 6 | 2 | 2 | 4 |  | 0 | 0 | – |
| Goat | Anhui | Liuan | 22 | 0 | 0 | 0 | 0 | 0 | 0 | 0 | 0 | 0 | 0 |  | 0 | 0 | – |
|  |  | Fuyang | 144 | 0 | 0 | 0 | 0 | 0 | 0 | 0 | 0 | 0 | 0 |  | 0 | 0 | – |
|  |  | Chuzhou | 110 | 0 | 0 | 0 | 0 | 0 | 0 | 0 | 0 | 0 | 0 |  | 0 | 0 | – |
|  |  | Bengbu | 18 | 0 | 0 | 0 | 0 | 0 | 0 | 0 | 0 | 0 | 0 |  | 0 | 0 | – |
|  |  | Anqing | 109 | 0 | 0 | 0 | 0 | 0 | 0 | 0 | 0 | 0 | 0 |  | 0 | 0 | – |
|  |  | Chizhou | 71 | 0 | 0 | 0 | 0 | 0 | 0 | 0 | 0 | 0 | 0 |  | 0 | 0 | – |
|  |  | Maanshan | 100 | 2 | 2.00 | 100 | 0 | 0 | 0 | 0 | 0 | 0 | 0 |  | 0 | 0 | – |
|  | Jiangsu | Xuzhou | 74 | 0 | 0 | 0 | 0 | 0 | 0 | 0 | 0 | 0 | 0 |  | 0 | 0 | – |
|  | Henan | Luoyang | 109 | 0 | 0 | 0 | 0 | 0 | 0 | 0 | 0 | 0 | 0 |  | 2 | 1.8 | CC1 |
|  | Shandong | Taian | 24 | 0 | 0 | 0 | 0 | 0 | 0 | 0 | 0 | 0 | 0 |  | 0 | 0 | – |
| Total | | | 781 | 2 | 0.3 | 100 | 0 | 0 | 0 | 0 | 0 | 0 | 0 |  | 2 | 0.3 | CC1 |
